# Resilience during Crisis and the Role of Age: Involuntary Telework during the COVID-19 Pandemic

**DOI:** 10.3390/ijerph19031762

**Published:** 2022-02-04

**Authors:** Susanne Scheibe, Jessica De Bloom, Ton Modderman

**Affiliations:** 1 Department of Psychology, University of Groningen, 9712 TS Groningen, The Netherlands; 2 Department of HRM & OB, University of Groningen, 9747 AE Groningen, The Netherlands; j.de.bloom@rug.nl; 3 Department of Psychology, Tampere University, 33014 Tampere, Finland; 4 Department of Health and Safety, University of Groningen, 9712 CT Groningen, The Netherlands; a.j.modderman@rug.nl

**Keywords:** coronavirus, work and age, resilience, well-being, remote work, job demands–resource model, lifespan development

## Abstract

We investigated the relationship between age, resilience, job demands and resources, and self-regulation in 1715 university employees during the COVID-19 pandemic (February 2021) by means of an online survey with closed and open questions. Correlation, regression, and qualitative analyses showed that older employees reported higher resilience than younger employees. This finding was robust after controlling for background factors (i.e., gender, expat status, job type, living alone). Age and resilience were directly related to higher job resources (i.e., job security and equipment), work–life balance, and seeing positives, whereas the relationship to demands was ambiguous. Age was unrelated to workload, negatively related to childcare, and positively to eldercare. Resilience was negatively related to workload but unrelated to childcare or eldercare demands. When all variables were combined to jointly predict resilience, age, job resources, and self-regulation resources predicted resilience, whereas demands (i.e., workload, childcare, and eldercare demands) did not. Our findings suggest that age-related advantages in well-being have persisted during the COVID-19 pandemic. Older workers were more likely to reframe the crisis and see it as an opportunity for personal growth. They possess and utilize resources in unique and beneficial ways, which could also benefit younger workers.

## 1. Introduction

When the COVID-19 pandemic hit in 2020, it changed the working lives of people around the world—for some employees temporarily and for many permanently [1,2]—and amplified inequalities [3]. In the Netherlands, schools, daycare centers, restaurants, and bars were closed in March 2020, and employees were urged to work from home as much as possible. The lockdown measures continued until the beginning of May 2020, after which some measures were slowly loosened. After a summer with relatively normal working conditions (e.g., most companies allowed employees to work in their office several days a week), a second partial lockdown began in October 2020 and measures became increasingly stricter until society slowly began to open up again in April 2021. Like many other organizations, Dutch universities followed the governmental guidelines and requested their employees to work from home during the lockdowns. In February 2021, when the current study was conducted, university employees had been largely teleworking for nearly one year and were allowed to work at university premises only with the approval of their supervisor and after formal registration in an online system. 

The fundamental changes in working conditions, along with other pandemic-related impacts on private life, constituted an unusual, long-lasting stressor for employees. Relatively older employees may be particularly vulnerable, as they face an increased health risk if they catch the virus and may feel less technologically competent [4]. Moreover, a recent rapid review found that older workers perceived remote work more negatively and reported more communication breakdowns and tensions between generations, which was likely exacerbated by stereotypes against older workers that were perpetrated by organizations and governments [4]. Yet, surprisingly, data on well-being collected during the early phases of the pandemic suggest quite the opposite—age advantages in emotional experience and daily resilience documented in pre-pandemic times appeared to persist under the conditions of the threat of COVID-19 [5,6,7]. Job loss, job insecurity, and turnover intentions were lower in older compared to younger workers [8,9,10]. Overall, the scarce existing evidence on age, working during the pandemic, and well-being suggests both challenges and benefits for older workers. Accordingly, we set out to further our understanding of if and how having a higher age supports people in coping with or even thriving throughout a major health crisis. 

More specifically, we addressed two research questions. Firstly, we examined whether employees’ ages were associated with resilience, as indicated by the maintenance of mental, cognitive, and social well-being during the COVID-19 crisis. We built on the prior literature suggesting that older employees may be more resilient than younger employees when facing daily threats to well-being [11]. Secondly, adopting the lens of the job demands–resources model [12], which distinguishes demands and resources inherent in work and non-work life domains, we examined the role of three groups of factors underlying the relationship between worker age and resilience—work and home demands, job resources, and personal resources in the form of self-regulation. Understanding age-related differences in employees’ resilience during pandemic-driven telework as well as sources of resilience in the work and personal context are important. These insights can help organizations to identify which employees are most vulnerable and may be in need of organizational support and intervention (see also [4]). 

### 1.1. Involuntary Telework and Resilience

Teleworking is not a new concept or new way of working. Telecommuting, remote work, distance work, virtual office, or home-based telework are different terms referring to the same concept—performing work tasks away from the employer´s premises in the context of an employment relationship, usually using information technology [13]. Alongside the rapid developments in IT solutions, telework drastically increased after the turn of the century, with around 10–30% of the European working population working from home at least some of the time [14]. In 2002, Bailey and Kurland [15] presented a concise overview of the expected effects of telework on the individual worker, the organization, and society, such as productivity gains, increased job autonomy, and less traffic congestion. Empirical studies could indeed show that telework was associated with higher supervisor-rated and objectively measured job performance, job satisfaction, lower turnover intent, and role stress (for meta-analyses, see [16,17]). However, there have also been studies showing increased work–family conflict due to permeable boundaries between work and private life [18], lower levels of trust [19], slower wage growth, and fewer career prospects [16]. Research has also shown that the effects of telework are often dependent on contextual characteristics, such as worker characteristics, characteristics of the work location (e.g., number of interruptions), and the frequency and intensity of telework [17]. 

It is important to note that prior findings on the consequences of teleworking mostly referred to scenarios where teleworking was voluntary, well-prepared (e.g., hardware, software, and ergonomic workstations were in place), and part-time. In contrast, teleworking during the pandemic happened suddenly, often full-time, and largely involuntary, as governments and organizations mandated their employees to work from home whenever possible to contain the spread of the virus. This was also the case for universities that, rather unprepared, quickly moved to online modes of teaching, research, and management. Accordingly, forced telework during the pandemic could be expected to threaten employees’ well-being. In fact, the pandemic has been characterized as a career shock, an unexpected and adverse event caused by factors outside of employees’ control, which impacted employees’ daily work and career outlook [20]. 

Still, not everyone will be equally affected by adverse events in their everyday functioning and well-being. The concept of resilience captures individual differences in the way people are able to manage adversity and respond to crises. Resilience has been conceptualized in various ways in the organizational literature, for example, as a resource, ability, trait, process, or outcome [21]. Given these diverse conceptualizations, there is no standard way to frame and assess resilience. Studies that conceptualize resilience as a resource (often measured through self-report scales, such as the Connor–Davidson Resilience Scale [22]) have revealed that resilience resources can support people through crisis. Resilience is linked to lower burnout and higher work engagement, better mental health, and increased social support [23]. Recent evidence on resilience as a resource during the COVID-19 crisis is inconclusive, with some studies showing that some health care workers became more and some less resilient during the pandemic [24]. 

In this study, we took a complementary perspective on resilience and framed it as an outcome for employees when faced with adverse events. Specifically, we conceptualized resilience as the degree to which employees are able to maintain well-being and have positive experiences during times of adversity [25,26]. To capture resilience as an outcome, we defined it as feeling mentally healthy, cognitively sharp, and socially integrated during the pandemic-induced teleworking period, relative to pre-pandemic times. Thus, employees who are forced to work from home during the pandemic would be considered low in resilience if they suffer from reduced mental health, have difficulties concentrating on their work tasks, and feel socially isolated when comparing themselves with pre-pandemic times. In contrast, employees would be considered high in resilience if they feel at least equally mentally healthy, able to focus during worktime, and as socially integrated as they did before the pandemic. The three positive states of mental health, good cognitive functioning, and social integration correspond to three important aspects of successful aging according Rowe and Kahn [27]. The goal of the current study was to better understand individual differences in resilience as a function of employees’ ages. 

### 1.2. The Role of Employee Age

To understand age-related differences in resilience during forced telework, it is useful to adopt a lifespan developmental perspective [4,28,29,30]. According to this perspective, development occurs throughout adulthood and leads to a changing dynamic of gains and losses in different functional domains, such as cognition or self-regulation [31]. Moreover, the social contexts of individuals change as they move through different life stages. For example, young children may be present in the family in the earlier stages of adulthood, a higher workload may result from adopting a leadership position in midlife, and eldercare demands may arise for many older workers. These aging-associated changes in persons and their private and work contexts can lead to differential impacts of pandemic-driven telework for employees of different ages [4,30]. 

There is substantial evidence that, by and large, older adults enjoy higher emotional well-being relative to younger adults. For example, research with community samples suggests that older (vs. younger) adults generally tend to enjoy good mental health [32] and emotional well-being [33,34]. Among groups facing serious threats to well-being, such as cancer patients, older adults were found to report better affective states than younger patients [35]. In samples of employees, there is evidence of more positive affect and higher affective stability at higher ages [36,37]. Especially relevant to the present study, older workers have been found to experience higher affective well-being and attentional focus when experiencing daily work stress compared to younger workers [11]. Recent data from the pandemic confirms an age-related advantage in affect [5], distress [38], and managing daily stress [6,7]. With the current study, we aimed to examine whether age-related advantages in levels of well-being can also be found in employees who are forced to switch to telework during the COVID-19 pandemic. 

To explain the relationship between age and resilience, a lifespan perspective points to several potential mechanisms, including changes in aging individuals themselves and in their environment [30]. To organize these mechanisms, we adopted the lens of the job demands–resources (JD–R) model [12]. According to the JD–R model, work outcomes are generally predicted by demands and resources inherent in the workplace (i.e., environmental factors), and personal resources in the form of traits and skills that support effective coping (i.e., individual factors). Job demands are work conditions that cost employees effort and consume their energy, such as a high workload. Job resources are aspects of the job that help workers meet their job demands and motivate them at work, such as social support and suitable work equipment (e.g., an ergonomic chair). In the context of telework, work and non-work domains closely interact, and multiple inter-role transitions can occur throughout the day [18]. To account for this close linkage between work and home, we adopt the extended JD–R model by Demerouti et al. [39], which includes demands and resources both in the work and private spheres of life. An example of a home demand is the workload arising from child- or eldercare demands. 

#### 1.2.1. Age-Related Shifts in Demands and Resources

Age-related differences in resilience during involuntary telework may result from shifting demands and resources as they are typical for an individual’s life and career stage [39]. Adulthood has been characterized as a succession through different stages (young, middle, and older adulthood), which are each associated with specific developmental goals, tasks, roles, and activities [40]. Career development theories hold that careers unfold across multiple stages, such as exploration, establishment, and maintenance, which are linked with typical career concerns and a shifting salience of roles both at work (e.g., protégé, mentor, leader, retiree) and outside of work (e.g., child, partner, parent; [41]). Demerouti and colleagues [39] combined lifespan and career-span perspectives to propose increasingly favorable constellations of demands and resources as young employees become middle-aged and eventually older employees. 

Younger employees are assumed to face high demands in the job and—after the transition to parenthood—also at home, while they have relatively low levels of resources at their disposal [39]. Being in the exploration and establishment stage, their developmental tasks at work comprise finding a place in the organization, mastering the assigned work tasks, and building up human and social capital. At the same time, young employees often hold temporary jobs and find themselves in subordinate roles with lower levels of autonomy and salary. When being forced to telework during the pandemic, it has been, therefore, likely that young employees have lacked sufficient equipment (such as a separate office in the house or an ergonomic chair) and information (such as knowing who to approach for different support needs) relative to older coworkers. Young employees are also more likely to have young children at home, which leads to high family demands, especially during government-enforced daycare and school closures. 

Middle-aged employees are also assumed to face high job demands, but in contrast to young employees, these would be counterbalanced by high levels of resources in both work and nonwork lives [39]. Being in the maintenance stage, their work roles would include nurturing a high level of expertise in their chosen occupation through skill development and training, and possibly taking on leadership roles and the associated responsibility for younger and older staff members. At the same time, middle-aged employees likely enjoy high levels of resources, including job autonomy and job security, as they have often moved into professional positions and permanent jobs, certainly at the university. These resources have likely buffered the adverse effects of pandemic-induced telework. Although childcare demands are still present, children are typically older and thus require less close supervision during school closures or canceled school lessons. 

Older employees are assumed to face average demands while enjoying high levels of resources both at work and home [39]. Being in the late maintenance phase, older employees are assumed to have achieved a good mastery of their work tasks and become more selective and skilled in shaping their job demands according to their interests [30]. Moreover, being in senior positions, they tend to enjoy high autonomy and job security through permanent employment contracts. Family demands also may be lower than in earlier stages of adulthood, as children are older and independent; although, older employees may increasingly care for their older parents (that is, eldercare). When switching to telework, it can be expected that the relatively lower levels of demands coupled with high levels of resources buffer the impact of changes in work procedures on the well-being of older employees. 

#### 1.2.2. Age-Related Improvements in Self-Regulation

In addition to demands and resources, a third group of influential factors in the JD–R model that likely support resilience are personal resources. Older employees may also have more access to these. Personal resources are individual dispositions and behaviors, such as positive self-evaluations and self-regulation skills, which help employees to fulfill their job and home demands and make good use of their resources [12]. Several scholars have suggested that resources such as emotion regulation capacity or behavior regulation strategies (e.g., goal engagement and disengagement) help older workers cope with pandemic-related challenges better than young workers [30,42]. However, these arguments were based on pre-pandemic data and still await empirical testing [4]. In the current study, we included two aspects of self-regulation that are highly relevant to resilience in the face of involuntary telework—managing the work–nonwork interface (indicated by work–life balance and boundary strength) and emotion regulation in the form of seeing positive aspects of the pandemic (i.e., a form of positive reappraisal). 

Work–life balance refers to employees’ perceptions that different life domains (e.g., work and family) are in balance and that their various role-related expectations can be met [43]. A major predictor of work–life balance is the absence of work–life conflict, which can take on multiple forms [44]—time-based (when spending time in one domain interferes with time in the other domain), strain-based (when strain experienced in one domain undermines functioning in the other domain), or energy-based conflict (when fulfilling demands in one life domain undermines energy resources left for the other domain). One of the major drawbacks of telework is that work and nonwork life occur in the same physical space, which can undermine work–life balance by leading to frequent boundary transitions [18]. At the same time, employees have an active role in shaping the boundaries between work and non-work life spheres [43]. Several pre-pandemic studies have uncovered a positive relationship between age and work–life balance [45]. This may partly be due to a more favorable constellation of work/home demands and resources, as described above. Yet, controlling for such contextual factors, older workers were found to also actively create stronger boundaries that shield work from non-work life, and non-work life from work [46]. Stronger boundaries, in turn, were found to contribute to older employees’ lower work–life conflict and higher work–life balance compared to younger employees. 

Emotion regulation refers to people’s cognitive and behavioral strategies to modify the nature, intensity, and duration of their emotional experiences [47]. A prominent cognitive emotion regulation strategy is positive reappraisal—the reinterpretation of (negative) events in positive terms. For example, people may think that telework during the pandemic helps them slow down and reconsider their life priorities, or provides them with more time to spend with household members [26]. From a lifespan perspective, people become more motivated and skilled in regulating their emotions as they age [28]. A higher motivation to regulate emotions is thought to arise from shifts in time perspective that lead older employees to see their time at work as more limited and orients them to the importance of high emotional well-being in the present as opposed to the future [48]. Greater life experience also provides older employees with more expertise and skills in meeting emotional demands at work and home; for example, they tend to rely on more adaptive emotion regulation strategies, such as positive reappraisal [49]. Seeing positive aspects in adverse situations is an effective strategy to maintain well-being, especially in uncontrollable situations such as the pandemic. Taken together, older employees may have achieved higher levels of resilience than younger adults in the forced transition to telework during the pandemic, as they could rely on personal resources of work–life balance and positive reappraisal. 

### 1.3. The Present Study

In order to examine the role of age in employees’ resilience during pandemic-induced telework, we analyzed data from an employee survey among the staff of a large Dutch university. The survey addressed all staff—from teaching and research to management and support staff—and was conducted in February 2021, a time when employees had been involuntarily teleworking for nearly one year. The study allowed us to investigate two research questions. First, we examined whether age predicts resilience—herein operationalized as an outcome during times of adversity such that employees feel comparatively mentally healthy, cognitively sharp, and socially integrated as before the pandemic. In line with prior research findings of age-related advantages in mental health, emotional well-being, and daily stress resilience, we predicted a positive association between age and resilience. In examining age differences, we also paid attention to the robustness of findings across different employee groups. Earlier accounts have emphasized substantial heterogeneity in the outcomes of teleworking [16] and work outcomes of younger versus older workers more generally [4,30]. Given that young, middle-aged, and older employees are rather heterogeneous groups, we examined to what extent age-related benefits in resilience are robust across different demographic groups that are relevant in the current university environment. Specifically, we tested for the moderating role of gender, expat status (Dutch vs. international), function (academic vs. management/support staff), and household composition (living alone vs. with other household members). 

Second, we examined sources of resilience in order to provide insight on mechanisms relating age to resilience during the pandemic. As described above, employee age is thought to affect well-being through shifts in work/home demands and resources [39], as well as improvements in self-regulation [46,49]. Thus, we considered the role of demands, resources, and self-regulation in the age–resilience relationship. Specifically, we considered age-related differences in three work/home demands (childcare, eldercare, workload), three work resources (work equipment, access to information, job security), and self-regulation (work–life balance, positive reappraisal). We expected to find positive relationships between employee age and job resources as well as self-regulation. Correspondingly, we expected to find negative relationships between age and job demands. Although the cross-sectional nature of our data precluded testing causal effects, we approached this question by regressing resilience on both age and potential mechanisms. Finding that age effects are reduced or even disappear when accounting for demands, resources, and self-regulation would be consistent with the notion that these three groups of factors play a role in linking age with resilience. 

These research questions were addressed with the help of a survey with quantitative scales. In addition, we included several open questions to gain a deeper understanding of the underlying processes and reasons why and how people´s demands and resources have changed during the pandemic. 

## 2. Design and Methods

### 2.1. Participants

The participants included 1715 employees at a Dutch university who completed an employee survey on working from home and well-being during the COVID-19 crisis. The survey was distributed by the Board of the University to all 6541 employees via an internal mailing list and was open from 1–9 February 2021. At this time, the Netherlands was in partial lockdown and the university requested all their employees to work from home as much as possible. Approval from the direct supervisor and registration through an online system were needed to enter the university buildings. Completing the survey was voluntary and anonymous; participants gave separate consent for the processing of their data for internal purposes (i.e., informing the board about what can be done to enable employees to work from home and stay healthy and motivated) and for research purposes. 

Of the 2032 survey respondents (response rate of 31%), 1715 (84%) consented that their data can be used for research purposes. The final sample was 62% female and 38% male; in terms of age, 5% were 25 years and younger, 31% were 26–35 years, 24% were 36–45 years, 22% were 46–55 years, and 18% were 56 years or older. About two-thirds (77%) were Dutch nationals, while 23% were non-Dutch internationals. In terms of employment contracts, 28% had a temporary contract, while 72% had a permanent contract. The sample comprised 48% academic staff (professors, researchers, and teachers) and 52% management and support staff (e.g., librarians, receptionists, facility managers, HR professionals, financial managers, communication professionals, legal professionals, student assistants, etc.). In terms of living arrangements, 24% reported living alone, while 76% lived with other household members. 

To estimate the sample’s representativeness for the parent population (i.e., staff of the university), we compared the demographic characteristics with the overall workforce of the university at the time of the survey, provided by the Human Resources department. The comparison revealed that our sample was largely comparable to the parent population in terms of staff type (academic vs. support staff), expat status, and age composition (except for the youngest age group, which was underrepresented in our sample with 5% vs. 14% in the parent population). Men were underrepresented (37% vs. 48% in the parent population) relative to women (59% vs. 52%), and staff with a temporary contract were underrepresented (27% vs. 50%) relative to staff with a permanent contract (68% vs. 50%). We return to the issue of sample representativeness in Section 4.

### 2.2. Measures

The survey could be completed in Dutch or English and contained a mixture of closed and open-ended questions. Owing to the purpose of the study as an employee survey (i.e., reducing respondent burden to reach a large number of staff members and leaving room for nuanced responses to the open questions), we measured nearly all constructs with single-item measures. The use of single-item rating scales is well established in large-scale epidemiologic surveys, and their test–retest and predictive validity have been demonstrated (e.g., for mental health—[50], for work–life balance—[51], for workload—[52]). In Section 2.2.1, Section 2.2.2, Section 2.2.3 and Section 2.2.4, we describe the measures relevant for the current set of analyses.

#### 2.2.1. Resilience

Consistent with our definition of resilience as feeling mentally healthy, cognitively sharp, and socially integrated (see [27]), we operationalized resilience by averaging responses to three single-item measures, respectively referring to mental health (“Compared to the months before the COVID-19 outbreak, your mental health is (1) much worse to (5) much better”), attentional focus (“Compared to the months before the COVID-19 outbreak, how much effort does it take you to concentrate during the working day—(1) much more effort to (5) much less effort”), and social integration (“Compared to the months before the COVID-19 outbreak, how often do you feel lonely—(1) much more often to (5) much less often”). A value of 3 referred to equal levels before and after the COVID-19 outbreak. Responses to the items on attentional focus and social integration were reverse-coded. Please note that although the survey was cross-sectional in nature, the wording of these items required participants to indicate the *perceived change* in their functioning relative to the time period before the COVID-19 pandemic. The internal consistency of the 3-item resilience measure was satisfactory (Cronbach’s α = 0.72).

#### 2.2.2. Work and Home Demands

Work demands were assessed on a single item (“Compared to the months before the COVID-19 outbreak, my workload over the past three months has been (1) much lower to (5) much higher”). Home demands were assessed on two dichotomous items, childcare demands (1 = living with children 12 years or younger; 0 = not) and eldercare demands (1 = caring for sick or disabled relatives, friends, or acquaintances; 0 = not). 

#### 2.2.3. Work Resources

Two dichotomous items assessed sufficient technical resources (I have sufficient resources (such as a PC, desk, stable internet, etc.) to be able to do my work well at home”—1 = yes, 0 = no) and information (“I have sufficient information to be able to carry out my work at home (information from your supervisor, Faculty Board/Director, Board of the University, the university website)”—1 = yes, 0 = no). Job security was operationalized as having a permanent (coded 1) as opposed to a temporary contract (coded 0). 

#### 2.2.4. Self-Regulation and Open Questions

Two open-ended questions were asked about work–life boundary management and seeing the positive sides of the pandemic. These were preceded by closed questions, after which the participants were asked to explain their answer (“Compared to the months before the outbreak of COVID-19, my work–life balance is (1) much worse to (5) much better. Please explain your answer.” And “Has working from home during the COVID-19 pandemic also had any positive effects? (1 = yes, 0 = no). Which positive effects would you like to mention/keep?”). 

### 2.3. Qualitative Analysis Procedure

We followed a conventional qualitative content analysis approach, where codes emerge from the data [53]. Five trained student research assistants coded the open responses. In a first step, one of the research assistants read through the responses of 100 participants to a given question and identified an initial list of categories that captured the responses. These lists of categories were then discussed and fine-tuned by the students and the coauthors. This resulted in a list of 20 categories (grouped under 6 higher-order categories) for the work–life question and 22 categories (grouped under 5 higher-order categories) for the question of the positive effects. Table 1 lists all the categories. 

In a second step, three student assistants tested the coding scheme by coding the same 100 responses independently. During the coding, multiple categories were allowed, as some participants reported multiple experiences. The resulting interrater agreement (Krippendorf’s Alpha) was satisfactory, with 0.82 for the question on work–life balance and 0.94 for the question on seeing positives. An additional meeting was held among the students and the coauthors to make some adjustments to the coding scheme where necessary. At this point, two new students were added to the team to manage the large amount of data. The two new students practiced the coding scheme with a new set of 100 responses. The resulting interrater agreements between the two new students and one of the initial research assistants were 0.98 and 0.84, respectively, for the two questions. 

In a third and final step, the remaining material was split between the five research assistants and coded individually. 

## 3. Results

### 3.1. Age Differences in Resilience

**Figure 1:**
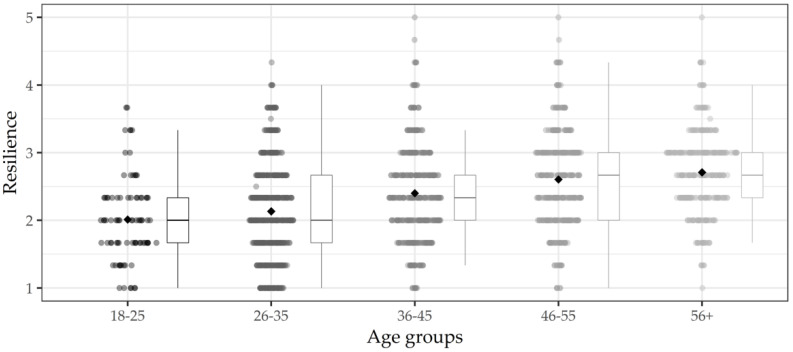
Boxplot of resilience scores across age groups. The response scale ranged from 1 = much worse to 5 = much better, with 3 = no change (relative to the months before the COVID-19 pandemic).

Figure 1 displays resilience scores for the five successive age groups. Resilience was lowest in the youngest age group and successively higher in the older age groups. The bivariate correlation between age and resilience was *r* = 0.32 (*p* < 0.001). Notably, the figure also indicates heterogeneity within the age groups. In each age group, some individuals reported higher well-being in the midst of the pandemic when comparing themselves to before the pandemic (i.e., scores above the scale midpoint of 3.0). Moreover, the figure indicates that the heterogeneity within age groups increased with age, with the older three age groups showing a larger spread of scores across the response scale than the two younger age groups.

To test the robustness of age differences in resilience across the genders, staff groups, expat status, and living situation, we used Hayes’ [54] PROCESS Macro for SPSS (Model 1). We ran separate moderation models for each potential moderator. Age differences in resilience were not moderated by gender (*b* = 0.027), staff group (*b* = 0.056), or expat status (*b* = 0.032, all 95% CIs included 0, all *p*s > 0.05). However, age differences were moderated by living situation (*b* = 0.090, *p* = 0.007), such that age differences were larger in employees living alone (effect = 0.245, 95%CI (0.190, 0.301), *p* = 0.001) than in employees living with other household members (effect = 0.156, 95%CI (0.123, 0.189), *p* = 0.001). In fact, differences between individuals living alone were largest in the youngest age group (Cohen’s *d* = −0.50) and smallest in the oldest age group (Cohens’ *d* = 0.00). 

In addition, all four moderator variables had unique associations with resilience after accounting for age (gender: *b* = 0.080, 95%CI (0.010, 0.149); staff group *b* = −0.358, 95%CI (−0.425, −0.291); expat status *b* = −0.232, 95%CI (−0.318, −0.146); living situation *b* = −0.189, 95%CI (−0.270, −0.209); all *p*s < 0.001). Specifically, resilience was lower in men (*M* = 2.35, *SE* = 0.03) compared to women (*M* = 2.43, *SE* = 0.02), in academic staff (*M* = 2.22, *SE* = 0.02) compared to management/support staff (*M* = 2.58, *SE* = 0.02), in expats (*M* = 2.22, *SE* = 0.04) compared to Dutch nationals (*M* = 2.46, *SE* = 0.02), and in employees living alone (*M* = 2.24, *SE* = 0.04) compared to employees living with other household members (*M* = 2.46, *SE* = 0.02). 

### 3.2. Correlational and Regression Analyses

Table 2 lists descriptive information of the quantitative variables along with their intercorrelations. Age was related to the three types of demands in a nonsystematic manner; there was a negative correlation with childcare demands, a positive correlation with eldercare demands, and no relationship with workload. The three types of demands, in turn, also showed nonsystematic relationships with resilience. Only workload was correlated with resilience in the expected negative direction (i.e., higher workload predicted lower resilience), while childcare and eldercare demands were unrelated to resilience. Regarding resources, age was positively related to job security and equipment, and all three resources (job security, equipment, and information) were positively related to resilience. Among self-regulation variables, age was positively related to work–life balance and seeing positives, which in turn, were positively related to resilience. These correlations hint at job resources and self-regulation—but not demands—as possible mechanisms underlying the age–resilience relationship. 

To further probe the shared variance between age and explanatory factors (demands, resources, and self-regulation) for the prediction of resilience, we performed a series of regression analyses. Table 3 contains the results of five models. Model 0 accounted for age only, Model 1 to 3 accounted for each group of predictors separately, and Model 4 accounted for all groups of predictors simultaneously. As can be seen in the table, each group of predictors added a significant amount of predicted variance (all Δ*R*^2^ are significant). The age effect was reduced when accounting for job resources and self-regulation (from *B* = 0.196 in Model *0* to *B* = 0.129 in Model 1 and *B* = 0.147 in Model 3), yet not when accounting for demands (*B* = 0.207 in Model 2). Moreover, among the demands, higher workload predicted lower resilience in Model 2, which accounted for demands only, yet workload no longer predicted resilience in Model 4, which accounted for all predictors simultaneously. Thus, none of the demands emerged as a robust predictor of resilience. It is further notable that even in Model 4, which accounted for all predictors simultaneously, age remained a significant predictor of resilience. Overall, the models suggest that part of the predictive effect of age on resilience can be accounted for by job resources and self-regulation.

### 3.3. Work–Life Balance (Qualitative Analysis)

The majority of participants (*n* = 1331; 77.6%) provided open-text responses to the question on work–life balance. The responses could be divided into six higher-order categories, denoting either negative changes (more work–life conflict, fuzzier work–nonwork boundaries, lack of social contact), positive changes (less work–life conflict, stricter work–nonwork boundaries), or no changes (work–life balance is good/stayed the same). Each higher-order category comprised 3–4 subcategories, with the exception of the no-change category, which had no subcategories. Table 1 lists the codes and a count of respondents whose answers reflected a given code.

Only a small number of responses (6 out of 100) indicated that *work–life balance is good or stayed the same*. For instance, respondents indicated that “at the beginning, it was a bit of a search, but now I’ve found my way.” (ID 694). The low frequency of this category demonstrates the profound impact of the pandemic on the work–nonwork interface.

The largest number of responses reflected more work–life conflict or fuzzier work–nonwork boundaries. Regarding *increased work–life conflict*, responses could be subdivided into strain-based conflict—“I am constantly mulling over work when I am trying to unwind.” (ID1715), time-based conflict—“I am only working to fulfill teaching duties, 7 days a week, with no vacations.” (ID144), energy-based conflict—“Private life exists less and less in my experience; therefore, recharging is less possible.” (ID49), or general conflict—“Work and private life get more mixed up.” (ID1668). Of the different types of work–life conflict, temporal conflict was noted twice as often (10 out of 100 respondents) than either strain- or energy-based conflict (4–5 out of 100 responses). Other responses referred to *fuzzier boundaries*, including fuzzier spatial boundaries—“My life now largely takes place at home, which means that ‘home’ is no longer a place where you can let go of work and relax. The home office keeps staring at you constantly.” (ID 571), fuzzier temporal boundaries—“It’s harder to stick to work hours; it’s easier to stay at the desk or do something (for work) in between when you’re free.” (ID409), fuzzier social boundaries—“During office hours, I am regularly disturbed by my children.” (ID232), or generally fuzzier boundaries—“It is next to IMPOSSIBLE to separate work and private life at the moment. We are expected to be always contactable, always online, or always available to get things done. We are not given enough time to relax and recover from our work.” (ID1155). As for work–life conflict, fuzzier temporal boundaries were reported most often (14 out of 100 responses), followed by fuzzier spatial boundaries (9 out of 100) or fuzzier social boundaries (4 out of 100). A few respondents also noted a *lack of social contact*, including a lack of work-related contact (2 out of 100 responses)—“Less satisfied with contact with colleagues; miss the real contact and the informal contacts.” (ID176), and non-work-related contact (less than 1 out of 100)—“I can’t meet friends, I can’t do the sports I would normally do. I spend the extra time working or thinking about work.” (ID 1110). 

Remarkably, there was also a smaller group who reported less work–life conflict or stricter work–nonwork boundaries during the lockdowns. For the category *less work–life conflict*, a good number of respondents (8 of 100) noted less time-based conflict—“No travel time provides 8 h a week of extra time for work and family.” (ID 194). Very few noted less energy-based conflict (1 of 100)—“Because I can work more quietly at home, I am less tired in the evening.” (ID 301), or less strain-based conflict (less than 1 of 100)—“Because my workday is less hectic, I have more mental resilience to value experiences.” (ID 1221). A small group also reported *stricter work–nonwork boundaries* (mostly in comparison to the first lockdown), including stricter spatial boundaries (1 in 100)—“I am lucky to have an independent room where I have a home office. Thus, I can switch between working/parent mode quite easily (most of the time).” (ID 1121), stricter temporal boundaries (2 in 100)—“I have been careful to keep my work time and my private time well separated; only once in a while do I go over the boundaries I have drawn.” (ID1336), or stricter social boundaries (less than 1 of 100)—“Better separation between private life and work, as there are now not many options to meet people from outside work.” 

To investigate age differences in the open responses about work–life balance, we performed two sets of analyses (see Table 4). First, we performed chi-square tests that took into account the five different age groupings and possible non-linear trends (e.g., 35–45-year-old employees may mention a certain category more often than all other age groups). Second, we performed logistic regressions predicting the likelihood of mentioning a given category as a function of age (used as a pseudo-linear variable). To ensure that the findings are meaningful, we performed both kinds of analyses only for the main categories, and only if these were mentioned by at least 5% of respondents. 

As can be seen in Table 4, both the chi-square test and the logistic regression yielded age differences in the categories no change in work–life balance and less work–life conflict. Both of these categories were mentioned more often by relatively older respondents compared to younger respondents. For example, less work–life conflict was mentioned by only 2.4% of the youngest age group but was mentioned by 13.9% of the oldest age group. No change in work–life balance was mentioned by only 1.2 % of the youngest group but by 10.9% of the oldest age group. The odds of mentioning no change in work–life balance were 1.35 times higher per increase of a decade; the odds of reporting less work–life conflict were 1.30 times higher per increase of a decade. 

For the category more work–life conflict, only the chi-square test was significant, while the logistic regression yielded a non-significant age effect. This was due to most work–life conflict being mentioned by the middle age group of 35–45-year-olds (39.2%). In comparison, more work–life conflict was mentioned by only 17.9% of the youngest age group and 21.2% of the oldest age group. 

### 3.4. Age and Seeing Positives (Qualitative Analysis)

The majority of respondents (*n* = 1382 or 82%) answered affirmatory to the question of whether working from home during the COVID-19 pandemic also had any positive effects. The responses could be divided into five higher-order categories (better work–life balance, better work conditions/productivity, healthier lifestyle, reflection/learning/personal growth, and other benefits), each with 4–6 subcategories. Table 1 lists the codes and a count of respondents whose answers reflected a given code. 

The most frequently mentioned benefit was *better work conditions/productivity*. In this broader category, a large number of respondents (19 of 100) noted a more flexible schedule—“I can do the work in my own rhythm without being disturbed.” (ID1335). Others reported that they could avoid stressors from the office environment (12 of 100)—“Office culture tends to help with maintaining a structure and healthy schedule, but also sometimes conveys a feeling of constant oversight and peer pressure to over-perform.” (ID825), better focus (11 of 100)—“I can do more work and I can work with more concentration.” (ID156), more effective (online) meetings (8 of 100)—“Large administrative meetings held online are more productive and quicker than in person; the attendance is higher, people are more focused.” (ID749), that it is possible to meet people located elsewhere (5 of 100)—“I can follow workshops/meetings abroad from home in which I otherwise would not participate.” (ID984), and generally better work conditions (7.5 of 100)—“Working from home is more fun than at the office; better ventilation; I can keep the dog company; only the chair is worse.” (ID185).

The second most frequently mentioned benefit was *better work–life balance*. Similar to the responses to the earlier question that asked specifically about work–life balance (see Section 3.3), the responses reflected less time-based conflict (35 of 100), less energy-based conflict (5 of 100), less strain-based conflict (2 of 100), and generally less conflict (3 of 100). Given that these categories overlapped with those in the prior section, we will not list specific quotes here. 

Another benefit was *reflection/learning/personal growth*. In this broader category, several people noted that they discovered new work methods (8 of 100)—“Online tools offer new flexibility and different opportunities when it comes to teaching, some of which I think could be incorporated in a hybrid way after the pandemic.” (ID1067). Others saw it as a time and impetus for reflection (2 of 100)—“Being grateful for things that, I think, most people took for granted, for example, contact with colleagues.” (ID757). A few people also noted that they learned new skills (1 of 100), “I became more confident at work, less dependent on colleagues for decision-making. I am a team player, but now I can also better make decisions myself.” (ID259). A handful (1 of 100) noted other learning/growth benefits—“It brought me and my girlfriend closer together, initially I had more time to talk with friends from my home country and I even started playing the guitar.” (ID782). 

Several responses reflected a *healthier lifestyle* due to working from home during the pandemic. These responses referred to being able to walk or move more (5 of 100)—“The ability to take a break during the day to go for a run, do some yoga or meditate.” (ID867), healthier eating or better food or coffee (4 of 100)—“I ate cheaper and healthier because I could prepare a delicious, nutritious, healthy lunch in my own kitchen. The university canteens, on the other hand, are unfortunately poor and expensive. This greatly benefited my concentration.” (ID1426), more or better sleep (1 of 100)—“I get more sleep because my ‘office’ is less than a minute from my bed.” (ID1155), or generally a healthier lifestyle (2 of 100)—“Creating healthier routines for eating, sleeping, exercising during the day, taking a break when needed and not feeling awkward about exercising, for example, laying on the ground to relieve my back.” (ID1082).

The final broader category was *other benefits*, which comprised less frequently mentioned benefits. A few respondents noted that they avoid commuting (in bad weather) (5 of 100)—“No more standing in long traffic jams due to commuting.” (ID1334), and that the pandemic let them live more environmentally friendly (2 of 100)—“less travel time and fuel needed, which is better for the environment.” (ID1230). Another benefit noted by a handful of respondents (2 of 100) was saving money—“I save money by being at home all the time and making all my own coffee/food.” (ID 775). Finally, some respondents noted other, more general benefits (4 of 100)—“I created a place of my own at home and I got to know colleagues in a partly different way, through different challenges and collaborations.” (ID654). 

To investigate age differences in the types of positive experiences reported, we again performed two sets of analyses (chi-square test, logistic regression) for all higher-order categories reported by at least 5% of the sample. The results are presented in Table 4. For two of the benefits, both the chi-square test and logistic regression yielded significant results—better work–life balance and a healthier lifestyle. Consistent with earlier results, older respondents were more likely to report a better work–life balance, with 40.1% in the oldest age group but only 22.6% in the youngest age group. The odds of reporting better work–life balance were 1.15 times higher with every additional decade of age. Surprisingly, health benefits were more often reported by younger employees than older employees (14.3% in the youngest age group vs. 7.3% in the oldest age group, with an odds ratio of 0.81). 

For two further categories, better work conditions/productivity and reflection/learning/personal growth, only the logistic regression—but not the chi-square test—yielded a significant age effect. The odds of reporting better work conditions/productivity were 1.094 times higher per decade of age, and the odds of reporting reflection/learning/personal growth were 1.172 times higher per decade of age. Overall, three of the six benefit categories were more often reported at relatively higher ages, whereas only one benefit (healthier lifestyle) was reported more at younger ages. 

## 4. Discussion

Since the COVID-19 pandemic started, thousands of jobs have disappeared, emerged, and changed drastically. Understanding who can cope with these major changes at work and which contextual factors foster resilience in crisis is essential. We used a survey with a combination of quantitative and qualitative questions to examine the relationship between age and resilience in a sample of 1715 university employees in the Netherlands. 

### 4.1. Main Findings

Concerning our first research question regarding age differences in resilience, we found that relatively older employees who are forced to telework showed higher resilience than younger employees one year into the pandemic. There was a linear increase in resilience with the oldest age group reporting the highest levels of mental health, ability to focus during worktime, and feeling socially integrated. The effect size can be considered large according to contemporary standards [55], with potentially powerful implications in both the short and long term. Age differences in resilience were robust across various subgroups of university employees, including men and women, Dutch and international staff members, as well as academic and management/support staff. Age differences in resilience were only moderated by the living situation such that age differences were larger in employees living alone than in employees living with other household members. In fact, among younger employees, living alone was associated with much lower resilience than living with household members, while the living situation did not matter much for the resilience of older employees. Overall, then, we can conclude that age differences are robust across people with different backgrounds in this sample of Dutch university employees. These findings contribute to a growing evidence base that an age-related advantage in well-being persists in the face of the threat of COVID-19. Adding to earlier studies that focused on community samples [5,6,7,38] or older age groups consisting of mostly retirees [26], our evidence confirms that positive age effects are also found among working-age employees. At this point, it seems that at least in the short and medium term, younger age groups struggle more in the face of the pandemic than older age groups. 

Concerning the second research question on sources of higher resilience at an advanced age, we found that age and resilience were directly related to higher job resources (i.e., job security and equipment), work–life balance, and seeing positives, whereas the relationships to demands were somewhat ambiguous. Age was not related to workload, negatively related to childcare, and positively to eldercare. Resilience was only negatively related to workload but unrelated to childcare or eldercare demands. These findings match our hypotheses and align with earlier propositions on age, resilience, and changes in demands and resources across the life span. Older workers more often have permanent jobs, have more established professional networks, and are also more likely in a financial situation that enables them to buy proper work equipment. These factors are usually connected to high well-being and thriving during a crisis. For instance, preliminary evidence from surveys conducted during the pandemic suggests that having a dedicated workspace with a door that keeps noise and interruptions by family members or flatmates outside may help with better concentration and performance throughout the crisis [56]. The fact that age was also related to better self-regulation resources matches earlier research as well. Greater life experiences seem to equip older employees with better skills to deal with emotional demands and superior adaptive emotion regulation strategies [49]. Focusing on positive aspects to maintain well-being, especially in uncontrollable situations such as the pandemic, is an example of this positive reappraisal, which older workers successfully apply. Moreover, older employees’ tendency to create stronger boundaries between work and nonwork life domains [46] may come in handy during times of teleworking when boundaries get blurred and it may be difficult to maintain a good work–life balance [18]. It should be noted that although the bivariate association between seeing positives and resilience was positive, the regression coefficient turned negative once work–life balance was added to the model. We consider this flip in the direction of the relationships as a statistical artifact arising from the large overlap between seeing positives and work–life balance. In fact, the open responses to the question on seeing positives revealed many answers referring to improved work–life balance. Future research may use fewer overlapping measures of these two variables to better understand their unique contributions to resilience. 

When all variables were combined, age, job resources (permanent work contracts, good equipment, and sufficient information), and self-regulation resources (work–life balance, seeing positives) predicted resilience during the crisis, whereas demands (i.e., workload, childcare, and eldercare demands) did not play a role. Hence, even when accounting for other explanatory variables, age remained a robust predictor of resilience. This raises the question of which other sources of resilience exist in older workers can explain the positive relationship. Naturally, the selection of variables in the current study was limited. Work/home demands and resources and additional self-regulation strategies other than those included in this study may also contribute to higher resilience with age. Examples of relevant contextual factors are job autonomy and sharing of responsibility among family members, social job resources, or the absence of social conflicts at both work and home. Examples of relevant personal factors are emotional labor, conflict management strategies, action regulation strategies, or more general disposition, such as self-esteem and self-efficacy [57,58,59]. Including additional potential demands, contextual resources, and personal resources—as implied by the JD–R model [12]—will help to further disentangle why younger employees may struggle more with the pandemic and forced teleworking than relatively older employees. 

### 4.2. Additional Findings

Two additional sets of findings are noteworthy. First, apart from age, our analyses revealed other demographic predictors of resilience. After accounting for age, male gender, being an international employee, being an academic (rather than support staff), and living alone were each uniquely related to lower levels of resilience. These results point at subgroups of employees who are particularly vulnerable. Given that all these demographic factors had unique predictive effects on resilience, certain combinations may be particularly problematic. In fact, a young international male academic employee (e.g., a Ph.D. student from abroad) living alone may be at particular risk for ill-being. In contrast, an older Dutch female manager living with other household members would most likely do rather well, and may even thrive during the crisis. In this sense, considering constellations of demographic variables helps in identifying subgroups of employees who need attention and interventions to prevent long-term mental health problems.

Second, our fine-grained analyses of open responses to questions on work–life balance and seeing positives revealed interesting nuances in the experiences of involuntary teleworkers that may easily be overlooked from quantitative measures. These responses also revealed the heterogeneity of experiences among employees. For example, employees differed greatly in their evaluation of the work–nonwork interface, with some employees noting negative changes (such as more time-based conflict, blurred boundaries), some employees noting positive changes (less time-based conflict), and others noting no changes to work–life balance. Older employees were more likely to spontaneously report no or positive changes than younger employees, which dovetails with the quantitative analyses. Regarding positive sides of the pandemic, a variety of effects were mentioned. These included better work conditions and productivity, learning and reflection, and a healthier lifestyle (e.g., preparing healthy home-cooked meals, exercising during the workday). Younger and older adults diverged somewhat in the positive effects that they experienced. Younger employees noted more often that they enjoyed a healthier lifestyle, whereas relatively older employees reported more benefits in terms of work conditions, work–life balance, and personal learning and growth. Among the whole sample, positive effects were mentioned by 82% of respondents. These encouraging findings attest to the overall strong resilience of individuals facing a major crisis. 

### 4.3. Theoretical Implications

The findings highlight the utility of adopting a lifespan developmental perspective when understanding resilience during the COVID-19 pandemic. Age seems to matter for employees’ experiences during this major health crisis, and age differences can be linked to changes in both aging individuals themselves as well as their work/home contexts. At the same time, our findings only partially confirm the lifespan and career stage perspective on the JD–R [39]. When pitching demands and resources against each other, resources had a much larger explanatory power for age differences in employee outcomes. This finding can also be interpreted in light of the fact that job resources usually have beneficial effects on occupational well-being, whereas the picture for job demands is more nuanced, sometimes showing beneficial and sometimes detrimental effects [60]. Therefore, researchers have recategorized demands into challenge and hindrance stressors (for a review, see [61]). Workload and caring for children or elderly parents may be examples of demands that can be both challenging and hindering.

Furthermore, our findings suggest that forced telework is much less beneficial than voluntary, well-prepared, part-time telework. This divergence from earlier studies on telework, which have shown mainly beneficial effects on well-being and performance (for reviews, see [16,17]), shows how influential and important the wider working context is. In order for beneficial effects to unfold, it is key that teleworking is voluntary, well-prepared (i.e., availability of hardware, software, and ergonomic workspaces to enable working remotely), and only part of the entire working week. Particularly for the on-boarding of new employees, creating a strong company culture, and trust, regular face-to-face interactions remain very important. 

### 4.4. Practical Implications

Our findings suggest important avenues for interventions. In particular, our results point to the importance of job security, equipment, and information for positive employee outcomes during periods of involuntary telework. These job resources can best be enhanced through group, leader, or organizational level interventions [62]. To increase job security, organizations should consider their employment contract policies. Educational institutions, such as universities, are operating on relatively stable markets and, thus, could afford to offer more employees permanent contracts sooner after organizational entry. Naturally, some job positions at the university are necessarily temporary (e.g., Ph.D. students, positions connected to external grants). In this case, organizations can consider contract extensions to help people bridge uncertain economic times. Organizations and leaders can further ensure that all employees have sufficient home office equipment. In cases where employees’ living arrangements do not provide an interruption-free space, special regulations should be designed to allow these employees to work on the employer´s premises. Informational resources may be enhanced through organizational, leadership, and team-level interventions. Organizations should invest in good knowledge management systems, such as webinars, well-organized intranet pages, or a centralized information desk that actively informs staff about possible support so that employees can easily find the information they need for their daily work. Teams can also develop ways to exchange knowledge. Each team member may have access to different types of information, and sharing this proactively with other team members may support the whole team’s well-being. Leaders also have a special role in ensuring that all employees receive the information they rely on in their daily work. Leaders should further show a leadership style that focuses on coaching and guiding (younger) employees, instead of a task-oriented approach. Our findings suggest that especially young employees would benefit from initiatives to enhance job resources and integration with the team. 

Apart from job resources, individual-level interventions may be well-suited to improve employees’ self-regulation. Short workshops, coaching sessions in peer groups, or individual coaching may be used to increase awareness of work–life balance and teach employees the importance of boundary management and emotion regulation. As being able to see positive sides of the pandemic was an important factor for resilience, it may be useful to actively stimulate positive reappraisals in employees, for example, in team activities. Creating diverse teams consisting of members of different ages may support intergenerational learning and enable younger employees to benefit from the experience and insights of older colleagues who may more easily see positive sides of this challenging situation. 

### 4.5. Limitations and Future Directions

One limitation of the current study is the reliance on self-reports, which introduces potential bias, especially in combination with the cross-sectional design. The concept of resilience is inherently temporal by nature, as it assumes the maintenance of well-being from pre- to post-event [21]. Although we asked participants to report on their changes in mental health, ability to focus, and social integration relative to pre-pandemic times, we still measured perceived rather than actual changes. Our study design, thus, made it difficult to fully test the assumed process model from age via demands, resources, and self-regulation to resilience. For example, it is possible that better well-being leads to more positive perceptions of demands and resources. Nevertheless, our model was derived from theoretical propositions and is generally backed up by longitudinal research showing prospective links between job characteristics measured earlier and well-being measured later [63]. 

Another limitation lies in our sample composition. Relative to the parent population, the youngest age group (under 25 years), men, and staff with a temporary contract were underrepresented. Given that these are exactly the groups that reported the lowest levels of resilience, we can conclude that staff who struggled more during the pandemic were less likely to participate in the employee survey. In turn, this suggests that our findings provide a slightly more positive picture of employee functioning than is warranted. Furthermore, participants were employees from one organization (a large public university) in one occupational sector (education). Although the educational sector was required to change its work procedures during the pandemic (moving from onsite to online modes of teaching and education), education continued throughout the pandemic. This is different from other sectors, such as the retail, catering, and tourism industries, where employees needed to cease their business operations for certain periods, or faced dramatic reductions in the number of customers. The university also represents a large organization, and employees in different types of organizations (small and medium-size businesses or self-employed) may have experienced the pandemic much differently. In fact, these other types of organizations may have experienced much higher levels of job insecurity than our current participants, which was an important predictor of resilience. Apart from business sector and type of organization, our sample was drawn from the Netherlands. Findings may, thus, not generalize to other countries. Notably, countries have differed quite substantially in terms of the stringency of their measures to control the pandemic. 

## 5. Conclusions

In our study involving 1715 Dutch university employees, we found that age was related to resilience during the COVID-19 pandemic, with higher mental, cognitive, and social well-being levels being related to advanced age. This finding was robust even after controlling for influential background factors, such as gender, expat status, job type, and living alone. As expected, based on prior literature, employees with many job resources (i.e., job security, proper equipment, and sufficient information) were more resilient during the health crisis than younger employees. In contrast, demands (e.g., childcare, eldercare) hardly affected resilience beyond age. Our analyses further revealed that older workers often reported enhanced work–life balance and less work–life conflict during the COVID-19 crisis. Older workers often even reported that their working conditions improved and that they were more productive than before the crisis. It seems that older workers were more likely to reframe the crisis and see it as an opportunity for personal growth. Older workers, thus, appear to possess and utilize resources in unique and beneficial ways, which could also benefit younger workers. Organizations could pay attention to providing job resources to all employees and supporting interactions between different generations of employees to foster social support and intergenerational learning. 

## Figures and Tables

**Table 1:** Codes for work–life boundaries and seeing positives.

Work–Life Balance Responses (*n* = 1331)	*n*	% ^a^	Seeing Positives Responses (*n* = 1382)	*n*	% ^a^
*1. Less work–life conflict*	*182*	*10.6*	*1. Better work–life balance*	*707*	*41.2*
1.1 Less strain-based conflict	7	0.4	1.1. Less strain-based conflict	30	1.7
1.2 Less time-based conflict	131	7.6	1.2. Less time-based conflict	592	34.5
1.3 Less energy-based conflict	17	1.0	1.3. Less energy-based conflict	84	4.9
1.4 Other/generally less conflict	35	2.0	1.4. Other/generally less conflict	57	3.3
*2. More work–life conflict*	*486*	*28.3*	*2. Better work conditions/productivity*	*785*	*45.8*
2.1 More strain-based conflict	70	4.1	2.1. More flexible schedule	327	19.1
2.2 More time-based conflict	190	11.1	2.2. Avoid stressors from office environment	203	11.8
2.3 More energy-based conflict	82	4.8	2.3. Better focus	194	11.3
2.4 Other/generally more conflict	170	9.9	2.4. More effective (online) meetings	135	7.9
			2.5. Can meet people located elsewhere	86	5.0
*3. Fuzzier work–nonwork boundaries*	*620*	*36.2*	2.6. Other/generally better work conditions	128	7.5
3.1 Fuzzier spatial boundaries	153	8.9			
3.2 Fuzzier temporal boundaries	244	14.2	*3. Healthier lifestyle*	*188*	*11.0*
3.3 Fuzzier social boundaries	60	3.5	3.1. More walking/moving	90	5.2
3.4 Other/generally fuzzier boundaries	220	12.8	3.2. More/better sleep	16	0.9
			3.3. Healthier eating, better food/coffee	70	4.1
*4. Stricter work–nonwork boundaries*	*70*	*4.1*	3.4. Other/generally healthier lifestyle	27	1.6
4.1 Stricter spatial boundaries	15	0.9			
4.2 Stricter temporal boundaries	36	2.1	*4. Reflection, learning, personal growth*	*196*	*11.4*
4.3 Stricter social boundaries	9	0.5	4.1. Time/impetus for personal reflection	35	2.0
4.4 Other/generally stricter boundaries	16	0.9	4.2. Discovered new work methods	136	7.9
			4.3. New skill learning	22	1.3
*5. Lack of social contact*	*47*	*2.7*	4.4. Other/general reflection, learning, growth	15	0.9
5.1 Work-related: colleagues, students	28	1.6			
5.2 Non-work related: friends, family, others	10	0.6	*5. Other benefits*	*216*	*12.6*
5.3 Other/general lack of social contact	10	0.6	5.1. Live more environmentally friendly, less traffic	42	2.4
*6. Work–life balance stayed the same/*			5.2. No commuting (in bad weather)	85	5.0
*is balanced*	*110*	*6.4*	5.4. Save money	31	1.8
			5.5. Other/general benefits	67	3.9

Note: Responses were coded at the level of subcategories. Responses could receive multiple codes. ^a^ Percentages are relative to the total sample (*N* = 1715).

**Table 2:** Descriptions and intercorrelations of central variables.

	*M* (*SD*)	% yes	Intercorrelations								
			1.	2.	3.	4.	5.	6.	7.	8.	9.
Age ^1^	3.17 (1.20)										
2. Resilience	2.40 (0.74)		0.32 ***								
3. Workload	3.66 (0.99)		0.03	−0.17 ***							
4. Childcare ^2^		24%	−0.08 ***	−0.01	0.07 **						
5. Eldercare		14%	0.20 ***	0.03	0.08 **	−0.05 *					
6. Job security		72%	0.54 ***	0.27 ***	0.05	0.12 ***	0.08 **				
7. Equipment		72%	0.12 ***	0.20 ***	−0.09 ***	−0.03	−0.06 *	0.04			
8. Information		94%	0.01	0.17 ***	−0.05 *	0.03	−0.05	−0.02	0.12 ***		
9. Work–life balance	2.37 (1.14)		0.15 ***	0.56 ***	−0.33 ***	−0.09 ***	−0.03	0.11 ***	0.20 ***	0.10 ***	
10. Seeing positives		82%	0.05 *	0.30 ***	−0.11 ***	0.05 *	0.01	0.06*	0.09 ***	0.10 ***	0.23 ***

Note: ^1^ Measured in terms of decades: 1 = 25 years or younger, 2 = 26–35 years, 3 = 36–45 years, 4 = 46–55 years, 5 = 56 years and older. ^2^ Coded 1 = children aged 0–12 years, 0 = no children aged 0–12 years. * *p* < 0.05. ** *p* < 0.01. *** *p* < 0.001.

**Table 3:** Results of stepwise regression analysis predicting resilience (*N* = 1715).

	Model 0: Age Only		Model 1: Job Resources		Model 2: Demands		Model 3: Self-Regulation		Model 4: All Predictors	
	*B* (*SE*)	*p*	*B* (*SE*)	*p*	*B* (*SE*)	*p*	*B* (*SE*)	*p*	*B* (*SE*)	*p*
Age	0.196 (0.014)	0.001	0.129 (0.017)	0.001	0.207 (0.015)	0.001	0.147 (0.012)	0.001	0.099 (0.015)	0.001
*Resources*										
Job security			0.246 (0.044)	0.001					0.212 (0.038)	0.001
Equipment			0.258 (0.038)	0.001					0.115 (0.033)	0.001
Information			0.513 (0.076)	0.001					0.343 (0.065)	0.001
*Demands*										
Workload					−0.127 (0.017)	0.001			0.005 (0.016)	0.772
Childcare demands					0.033 (0.039)	0.397			0.009 (0.034)	0.798
Eldercare demands					0.073 (0.052)	0.155			0.023 (0.043)	0.591
*Self-regulation*										
Work–life balance							0.306 (0.013)	0.001	0.296 (0.014)	0.001
Seeing positives							−0.340 (0.037)	0.001	−0.300 (0.039)	0.001
*F*	190.23		78.53		63.78		353.27		122.31	
*df*	1		4		4		3		9	
*p*	0.001		0.001		0.001		0.001		0.001	
*R* ^2^	0.102 ***		0.166 ***		0.137 ***		0.389 ***		0.420 ***	
Δ*R*^2^			0.069 ***		0.031 ***		0.287 ***		0.317 ***	

Note: *** *p* < 0.001.

**Table 4:** Age group differences in main category codes for work–life boundaries and seeing positives.

	*n* (%)						Chi-Square Test		Logistic Regression		
	Total Sample	18–25	25–35	35–45	45–55	55+	Value (df = 4)	*p*	Odds (Age)	Wald	*p*
	Work–life balance responses (*n* = 1331), ordered by frequency										
Fuzzier boundaries	620 (36.2)	39 (46.4)	197 (38.0)	125 (31.6)	131 (35.1)	116 (38.4)	8.895	0.064	0.968	0.575	0.448
More work–life conflict	486 (28.3)	15 (17.9)	130 (25.1)	155 (39.2)	108 (29.0)	64 (21.2)	38.086	0.001 *	0.987	0.078	0.78
Less work–life conflict	182 (10.6)	2 (2.4)	39 (7.5)	46 (11.6)	51 (13.7)	42 (13.9)	18.499	0.001 *	1.304	15.789	0.001 *
No change in work–life balance	110 (6.4)	1 (1.2)	29 (5.6)	19 (4.8)	27 (7.2)	33 (10.9)	16.473	0.002 *	1.346	12.451	0.001 *
	Positive experiences (*n* = 1382), ordered by frequency										
Better work conditions/productivity	785 (45.8)	37 (44.0)	226 (43.6)	172 (43.5)	181 (48.5)	153 (50.7)	5.86	0.21	1.094	4.763	0.029 *
Better work–life balance	707 (41.2)	19 (22.6)	183 (35.3)	190 (48.1)	182 (48.8)	121 (40.1)	35.961	0.001	1.153	11.57	0.001 *
Reflection, learning, personal growth	196 (11.4)	5 (6.0)	49 (9.5)	46 (11.6)	48 (12.9)	41 (13.6)	6.669	0.154	1.172	6.036	0.014 *
Healthier lifestyle	188 (11.0)	12 (14.3)	69 (13.3)	50 (12.7)	33 (8.8)	22 (7.3)	10.775	0.029 *	0.811	9.817	0.002 *
Other benefits (e.g., get to know neighborhood)	216 (12.6)	6 (7.1)	60 (11.6)	57 (14.4)	50 (13.4)	39 (12.9)	4.174	0.383	1.075	1.381	0.24

Note: Follow-up tests for age were only performed if at least 100 people reported the category. The chi-square test compared age as categorical variables, thus capturing non-linear age trends. The logistic regression treated the age group as a pseudo-linear variable, thus capturing linear age trends. * *p* < 0.05.

## Data Availability

Data can be made available upon request.
